# Minimally invasive sacrocolpopexy: efficiency of robotic assistance compared to standard laparoscopy

**DOI:** 10.1007/s11701-023-01799-1

**Published:** 2024-02-10

**Authors:** Nikolaos Evangelopoulos, Aude Nessi, Chahin Achtari

**Affiliations:** Women, Mother, Children Department (DFME)-Urogynecology Unit, Vaud University Hospital Center (CHUV), Av. Pierre-Decker 2, 1011 Lausanne, Switzerland

**Keywords:** Sacrocolpopexy, Robotic assistance, Da Vinci, Overall operative time

## Abstract

Minimally invasive abdominal sacrocolpopexy (SC) is the treatment of choice for symptomatic, high-grade, apical or multi-compartmental pelvic organ prolapse (POP), in terms of anatomical correction and treatment durability. Robot-assisted sacrocolpopexy (RASC) could be an attractive alternative to the gold standard laparoscopic sacrocolpopexy (LSC), for its ergonomic advantages in such a technically demanding procedure. However, it has not yet proven its superiority, consequently raising cost-effectiveness issues. Our primary objective was to assess if RASC can achieve better overall operative time (OOT) over LSC, with at least equivalent perioperative results. This was a single-center retrospective study including 100 patients (58 consecutive RASC cases and 42 LSC within the same time-period), with primary endpoint the OOT in both groups. Secondary results included complication rate, hospital stay, short-term anatomic results and OOT within and beyond the RASC learning curve. A multivariate linear regression was carried out for our primary outcome. The groups had comparable characteristics, except for BMI, which was lower in RASC group. The mean OOT was significantly lower in the RASC group (188 vs. 217 min, *p* ≤ 0.01), even after adjusting for possible confounders. Short-term anatomic results, complication rate, and blood loss were similar in the two groups. Mean hospital stay was significantly longer in the RASC group. Average RASC OOT was significantly shorter after the first 20 cases realized. This study demonstrated a significant reduction of OOT for RASC compared to LSC, with similar perioperative results, encouraging further use of the robotic technology for this indication.

## Introduction

Abdominal sacrocolpopexy (SC) is the gold-standard surgical treatment for advanced stage, apical, associated or not with other compartment, pelvic organ prolapse (POP). Minimally invasive techniques gradually replaced open surgery for this indication given their superiority in terms of postoperative morbidity and patient recovery [1]. Current published data show that both laparoscopic sacrocolpopexy (LSC) and robot-assisted sacrocolpopexy (RASC) are better than laparotomy, in terms of blood loss, hospital stay and complications rate, for an at least equivalent anatomic result [2, 3]. LSC has become widely available because it is a safe and efficient minimally invasive method, although technically challenging, with a steep learning curve. From this perspective, robotic assistance represents an attractive alternative, as its inarguable advantages over ergonomics along with 3D vision, could balance the technical complexity of sacrocolpopexy, rendering this surgical technique feasible for more surgeons, with no compromise in results. Although robotic assistance is an already widely implemented approach of sacrocolpopexy, it still needs to prove itself, especially in terms of efficiency and cost effectiveness [4]. A recent meta-analysis including 13 comparative studies and pooling results from over 2000 patients, found no difference in postoperative results or complications, but on the other hand, significantly longer operative time for RASC [5]. On the other hand, RASC seems to be clearly advantageous for teaching purposes, with easier incorporation of the sacrocolpopexy technique in residents training, without negative effect on operative time nor surgical outcome and a good learning curve [6].

The aim of our study was to assess the feasibility and the efficiency of the robotic assistance for the sacrocolpopexy, when compared to the gold-standard laparoscopic approach. Our primary hypothesis was that the technological advantages offered by the robotic system should eventually lead to reduced overall operative time compared to classic laparoscopy, for a technically demanding surgery like the sacrocolpopexy. Furthermore, we wanted to gather and compare descriptive data on perioperative outcomes for both RASC and LSC in our department.

## Methods

This was a single-center retrospective study including all eligible patients recruited in the Vaud University Hospital Center (CHUV) of Lausanne, Switzerland, from May 2012 to Dec 2018.

All patients having undergone minimally invasive access sacrocolpopexy, with recoverable data and with no stated objection to their use for study purposes, were included. We kept anonymized data in an Excel file. The study respected the data collection and storage requirements according to the relative Swiss federal legislation (ORH, articles 5 and 25) and the study project received approval from the designated ethics committee before commencement of data processing (study ID number 2017-00803).

We collected data for 105 patients, 63 having undergone RASC and 42 LSC. We had to exclude five patients of the robotic arm due to missing variables, not permitting accurate calculation of OOT, which is our primary endpoint. That leaves us with a final study cohort of 100 patients, 58 in the RASC arm and 42 in the LSC arm.

All the robotic and laparoscopic procedures were conducted by the same senior surgeon, or under his direct supervision. The DaVinci^®^ Robotic System was used for all robotic cases, with an upgrade from the Si version to the Xi^®^ version in April 2016. This translates into the use of the da Vinci Si^®^ version the first 20 RASC cases conducted in our department, replaced from the Xi^®^ for the 38 later ones. Both in RASC and LSC, two polypropylene meshes (anterior and posterior) are placed systematically, with the posterior’s dissection depth depending on its degree of prolapse. For the posterior compartment, an associated rectopexy or even a vaginal colpoperineorrhaphy can be associated, depending on the indication. We use absorbable polyfilament sutures for the mesh fixation in the vesicovaginal and rectovaginal spaces and non-absorbable polyfilament, for its fixation on the cervix and for the fixation of the two meshes on the promontory. We performed a concomitant subtotal hysterectomy in virtually all cases where applicable, but a uterine conservation remains a possibility, in condition that uterine pathology or symptoms are absent. Finally, a concomitant urinary incontinence procedure was performed if indicated. Prolapse stage complies with the POP-Q international classification.

The main endpoint for both groups was the overall operative time (OOT), defined as the time interval between incision and wound closure, for both RASC and LSC groups. Specifically for the robotic arm, we calculated the docking time (DT), defined as the time necessary to install the robot correctly into the surgical field, with the robotic arms properly connected in their port sites. Three robotic arms along with an accessory assistant trocar of 12 mm were used in all operations. In the DaVinci^®^ older version (Si), the trocar diameters were 12 mm, replaced by the 8 mm trocars in the later Xi^®^ version. The setup time (ST) was defined as the total surgical procedure time before console time. The different time landmarks of the surgical procedures like time of the incision, start/end of docking, start/end of console time, start of surgical wound dressing marking the end of the procedure, are kept systematically in digital form by the anesthesia team. Other variables examined and compared between the two groups of this study were eventual perioperative complications, hospital length stay and short-term postoperative results as described in the 6-week post-operative follow-up. The presence of > 1st degree prolapse according to POP-Q classification, symptomatic or not, was considered as a poor anatomic result. Complications were described by the system affected and ranked according to Clavien–Dindo classification.

For our descriptive analysis Welch’s ANOVA or Mann–Whitney *U* test was used to compare mean values, while Fischer or Chi-square test was used for qualitative variables. A linear regression was carried out for our primary outcome OOT and the explanatory variables surgical approach (RASC vs. LSC), concomitant adhesiolysis or adnexal treatment, associated UI surgery, BMI, history of abdominal and/or vaginal surgery, history of hysterectomy and finally, concomitant subtotal hysterectomy. Statistical significance of 5% was set for all results.

## Results

The two groups were comparable for main characteristics like age, parity and abdominal surgery history. Median BMI was lower in the RASC group (23.8 vs. 25.7, *p* = 0.037) (Table 1). All the patients had an apical prolapse, which was ranked of at least 2nd degree in the POP-Q classification, in 84.7% in the RASC group and in 81% in the LSC group. 87% of the patients had a concomitant subtotal hysterectomy, proportion similar in both groups. 10% in the RASC group and 2.4% in the LSC group had a prior hysterectomy, which was not statistically significant. Only 9 out of the 87 hysterectomized patients (10.3%) had an hysterectomy indication other than the POP (abnormal uterine bleeding, pelvic pain).

**Table 1:** Study population characteristics

	RASC (*n* = 58)	LSC (*n* = 42)	*n*	*p*	Test
Age (yo), mean, (SD)	54.4 (11.3)	55.9 (9.66)	100	0.47	Welch
BMI, median (Q25–75)	23.8 (22.2; 26.6)	25.7 (23.3; 30.0)	99	0.037	Mann–Whitney
Menopausal, *n*					
Yes	26 (45%)	23 (55%)	49	0.33	Chi^2^
No	32 (55%)	19 (45%)	51	–	–
Parity, median (Q25–75)	2.00 (2.00; 3.00)	2.00 (2.00; 3.00)	100	0.25	Mann–Whitney
Anterior compartment POP-Q stage, *n*					
0	1 (1.7%)	0 (0%)	1	0.8	Fisher
1	10 (17%)	4 (9.8%)	14	–	–
2	20 (34%)	17 (41%)	37	–	–
3	26 (45%)	19 (46%)	45	–	–
4	1 (1.7%)	1 (2.4%)	2	–	–
Apical compartment POP-Q stage, *n*					
1	9 (16%)	7 (17%)	16	0.58	Fisher
2	33 (57%)	19 (46%)	52	–	–
3	15 (26%)	15 (37%)	30	–	–
4	1 (1.7%)	0 (0%)	1	–	–
Posterior compartment POP-Q stage, *n*					
0	17 (29%)	6 (15%)	23	0.41	Fisher
1	24 (41%)	20 (49%)	44	–	–
2	13 (22%)	12 (29%)	25	–	–
3	3 (5.2%)	3 (7.3%)	6	–	–
4	1 (1.7%)	0 (0%)	1	–	–
Overall POP-Q stage, *n*					
2	21 (36%)	11 (27%)	32	0.58	Fisher
3	36 (62%)	29 (71%)	65	–	–
4	1 (1.7%)	1 (2.4%)	2	–	–
Associated posterior compartment treatment, *n*					
No	55 (95%)	38 (90%)	93	0.12	Fisher
VRR	2 (3.4%)	0 (0%)	2	–	–
RP	1 (1.7%)	4 (9.5%)	5	–	–
Associated IU surgery, *n*					
No	36 (62%)	29 (69%)	65	0.47	Chi^2^
Yes	22 (38%)	13 (31%)	35	–	–
Blood loss (ml), median (Q25–75)	75.0 (37.5; 150)	100 (100; 200)	40	0.18	Mann–Whitney
Blood transfusion, *n*					
No	24 (96%)	42 (100%)	66	0.37	Fisher
Yes	1 (4%)	0 (0%)	1	–	–
Complications Clavien–Dindo stage, *n*					
0	45 (78%)	35 (83%)	80	0.83	Fisher
1	10 (17%)	5 (12%)	15	–	–
2	2 (3.4%)	2 (4.8%)	4	–	–
3	1 (1.7%)	0 (0%)	1	–	–
Conversion to laparotomy, *n*					
No	58 (100%)	41 (98%)	99	0.42	Fisher
Yes	0 (0%)	1 (2.4%)	1	–	–
Urinary tract complications, *n*					
No	56 (97%)	41 (98%)	97	1	Fisher
Yes	2 (3.4%)	1 (2.4%)	3	–	–
H/o abdominal and/or vaginal surgery, *n*					
No	26 (45%)	20 (48%)	46	0.78	Chi^2^
Yes	32 (55%)	22 (52%)	54	–	–
H/o HE, *n*					
No	52 (90%)	40 (98%)	92	0.23	Fisher
Yes	6 (10%)	1 (2.4%)	7	–	–
Concomitant sHE, *n*					
No	9 (16%)	4 (9.5%)	13	0.38	Chi^2^
Yes	49 (84%)	38 (90%)	87	–	–
Indication of HE other than POP, *n*					
No	52 (90%)	39 (93%)	91	0.73	Fisher
Yes	6 (10%)	3 (7.1%)	9	–	–
Adhesiolysis or adnexal treatment, *n*					
0	15 (26%)	6 (14%)	21	0.16	Chi^2^
1	43 (74%)	36 (86%)	79	–	–
Hospital stay (days), mean (SD)	3.36 (1.27)	2.64 (0.958)	100	< 0.01	Welch
Postoperative anatomic result, *n*					
Poor	3 (8.6%)	1 (4.5%)	4	1	Fisher
Good	32 (91%)	21 (95%)	53	–	–

*HE* hysterectomy, *VRR* vaginal rectocele repair, *RP* rectopexy

The mean overall operating time (OOT) was 188 min for the RASC group, significantly lower than in the LSC group with a mean OOT of 217 min **(*p* < 0.01)**. In the RASC group, the mean total setup time was 21 min and the mean docking time was 7 min (Table 2).

**Table 2:** RASC time intervals

	Mean (sd)	Median (Q25–75)	Min	Max	*n*
OOT (min)	188 (42.7)	189 (156; 220)	94.0	290	58
Setup time (min)	21.4 (7.51)	20.0 (16.0; 25.0)	9.00	41.0	58
Docking (min)	7.18 (3.78)	6.00 (4.00; 10.0)	3.00	20.0	57

**Fig. 1:**
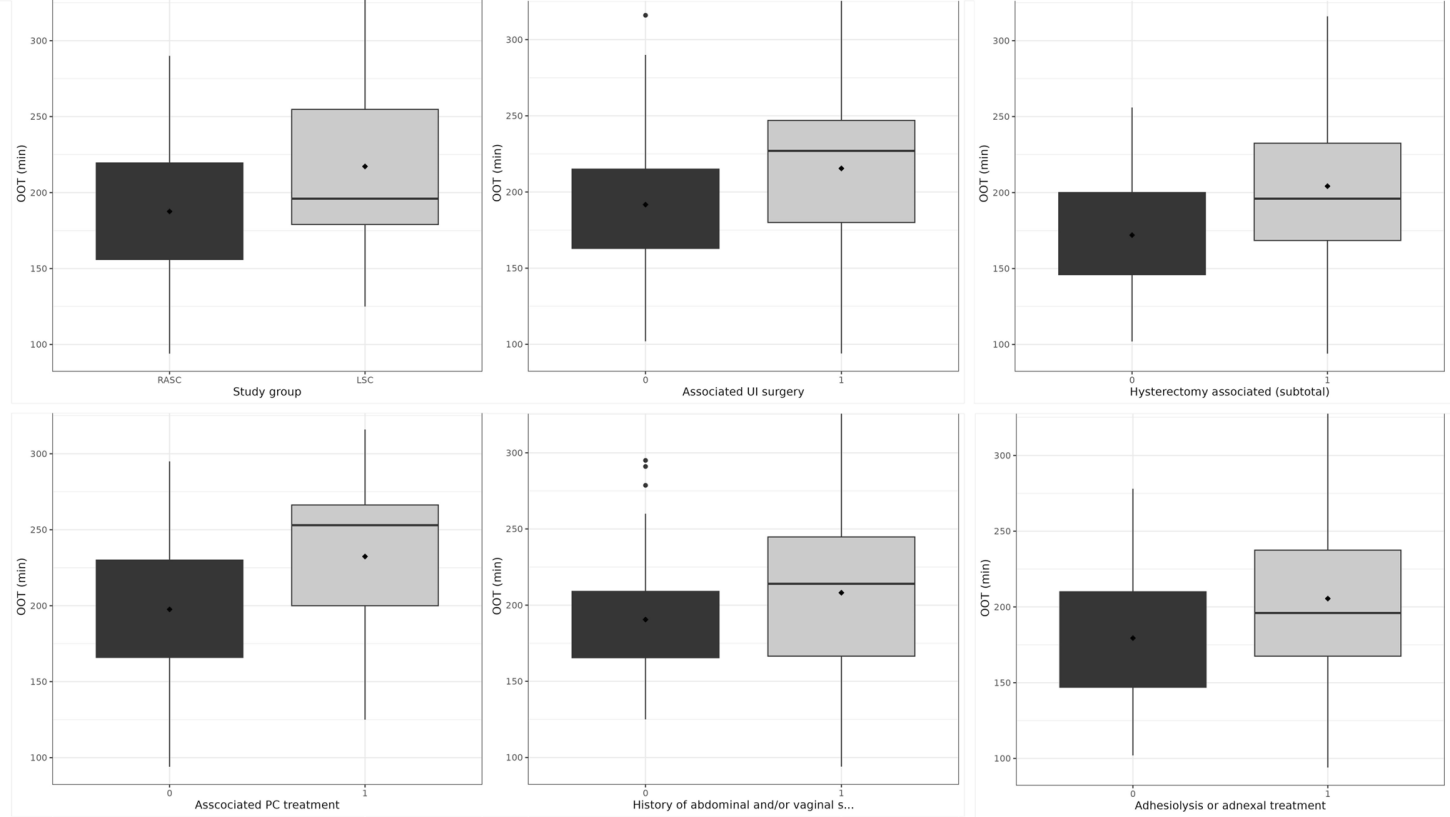
Box and whisker plot representing OOT difference between the two study arms as well as in different subgroups of interest

In the univariate analysis, OOT was significantly longer in the presence of adhesiolysis or adnexal treatment, concomitant urinary incontinence (UI) procedure or subtotal hysterectomy (sHE) (Fig. 1**, **Table 3).

**Table 3:** Univariate analysis for OOT in the study population (*n* = 100)

	Mean (sd)	Median (Q25–75)	Min	Max	*n*	*p*	Test
Study group							
RASC	188 (42.7)	189 (156–220)	94.0	290	58	< 0.01	Welch
LSC	217 (50.7)	196 (179–255)	125	331	42	–	–
Adhesiolysis or adnexal treatment							
No	179 (42.1)	182 (147–210)	102	278	21	0.02	Welch
Yes	206 (48.6)	196 (168–238)	94.0	331	79	–	–
Associated PC treatment							
No	198 (46.5)	190 ([166–230)	94.0	331	93	0.2	Welch
Yes	232 (62.8)	253 (200–266)	125	316	7	–	–
Associated UI surgery							
No	192 (42.0)	184 (163–215)	102	316	65	0.031	Welch
Yes	215 (55.6)	227 (180–247)	94.0	331	35	–	–
H/o abdominal and/or vaginal surgery							
No	191 (40.8)	183 (166–209)	125	295	46	0.056	Mann–Whitney
Yes	208 (52.9)	214 (166–245)	94.0	331	54	–	–
Concomitant sHE							
No	172 (45.6)	166 (146–200)	102	256	13	0.031	Welch
Yes	204 (47.5)	196 (168–232)	94.0	331	87	–	–

**Fig. 2:**
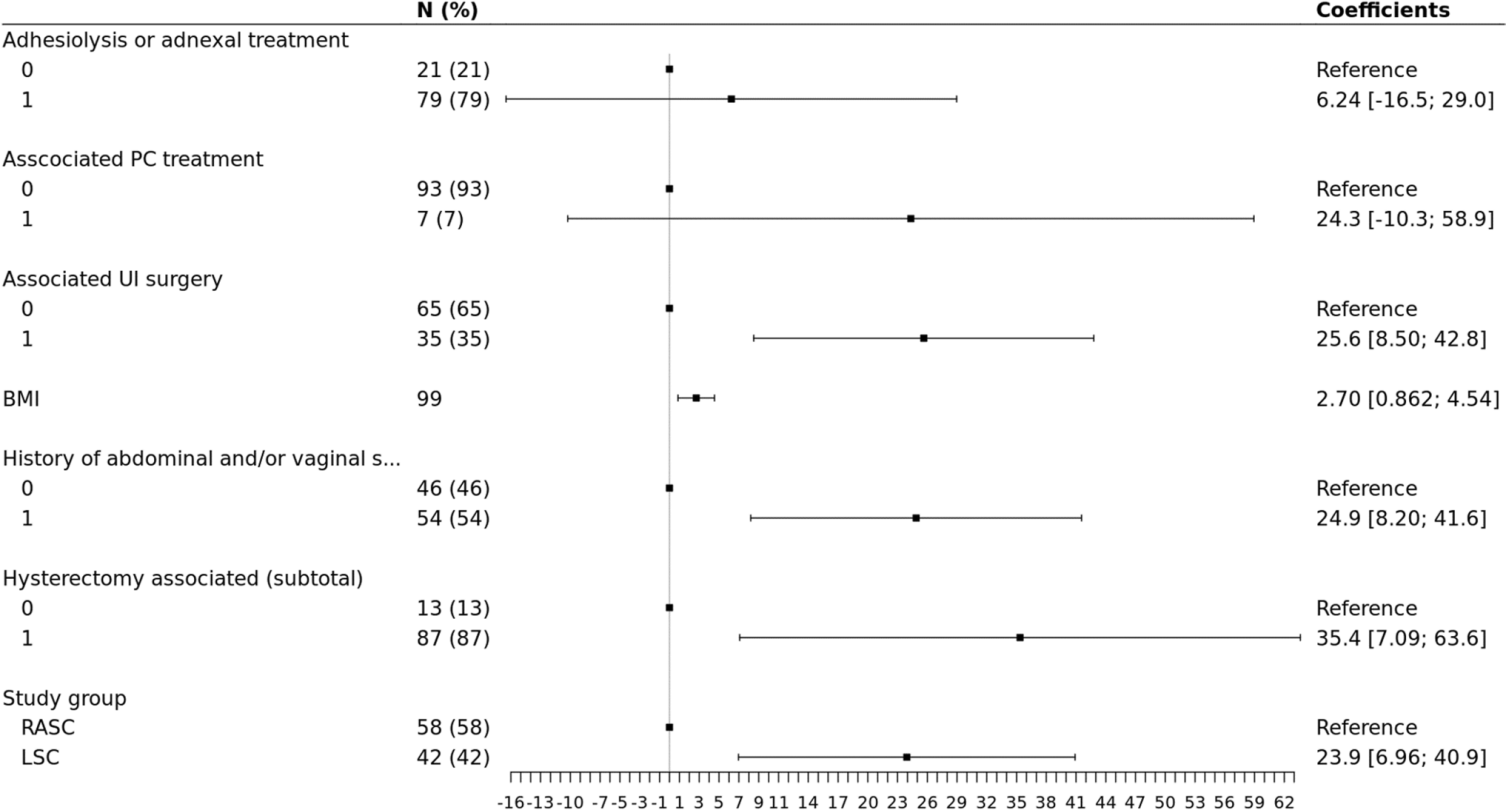
Forest plot based on the results of multivariate analysis of the factors associated with OOT differences

**Fig. 3:**
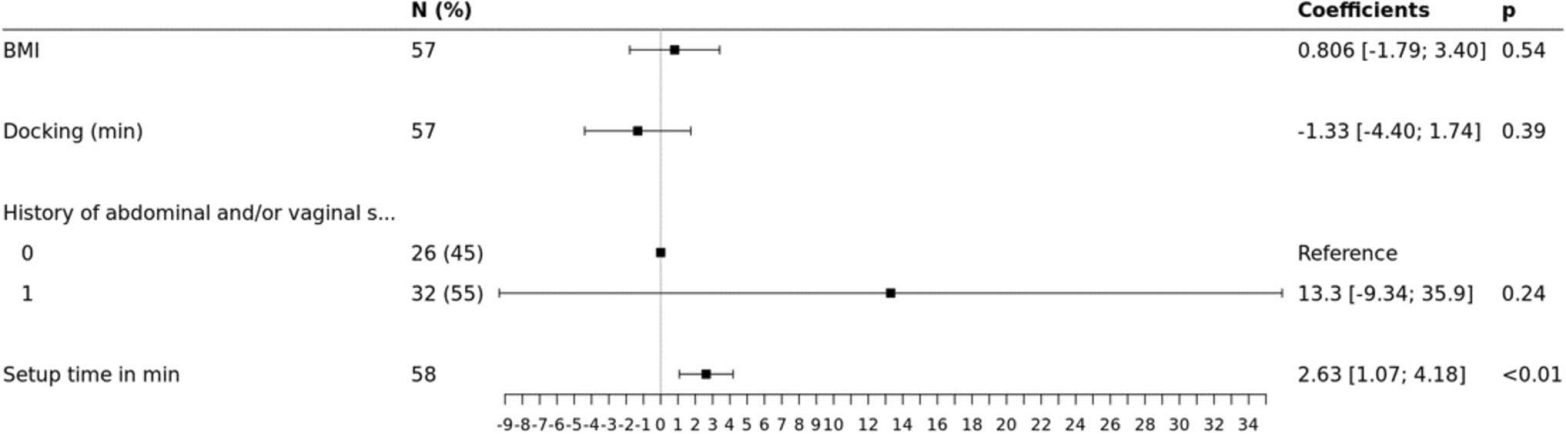
Forest plot of multivariate analysis of OOT for possible confounding factors, RASC group

In the multivariate analysis, by adjusting for adhesiolysis or adnexal treatment, associated posterior compartment (PC) treatment, associated UI surgery, BMI, history of (h/o) abdominal and/or vaginal surgery or concomitant subtotal hysterectomy (sHE), the difference of the OOT between the study groups remained statistically significant. The OOT in the group LSC was on average superior of 23.9 min compared to the group RASC (*p* ≤**0.01**). OOT was significantly linked to concomitant UI surgery (+ 25.6 min, *p* < **0.01),** BMI (increase by 1 unit(s) caused an average OOT increase of 2.70 min, *p* < **0.01),** h/o of abdominal and/or vaginal surgery (+ 24.9 min, *p* < **0.01**) and concomitant sHE (+ 35.4 min, *p* = **0.015**) when the total cohort was studied, (Fig. 2). However, when limited to the RASC group, BMI and h/o surgery where no longer significant (*p* 0.54 and 0.24, respectively), while setup time seems to significantly affect OOT (< 0.01) (Fig. 3).

**Fig. 4:**
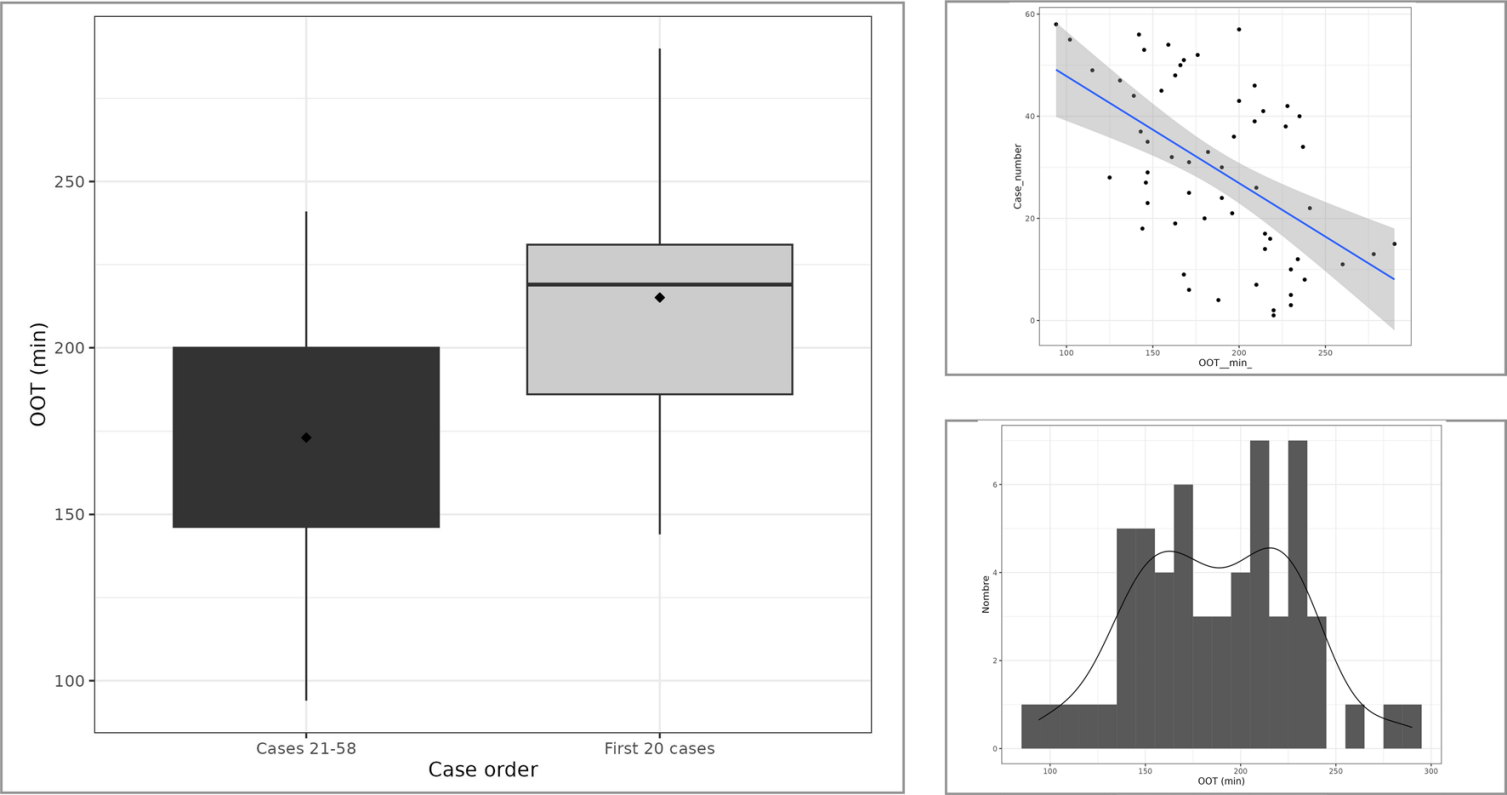
Box and whisker plot showing longer OOT within the learning curve first 20 RASC cases (**a**), **b** scatter plot representing this relationship, **c** OOT distribution for RASC cases

**Fig. 5:**
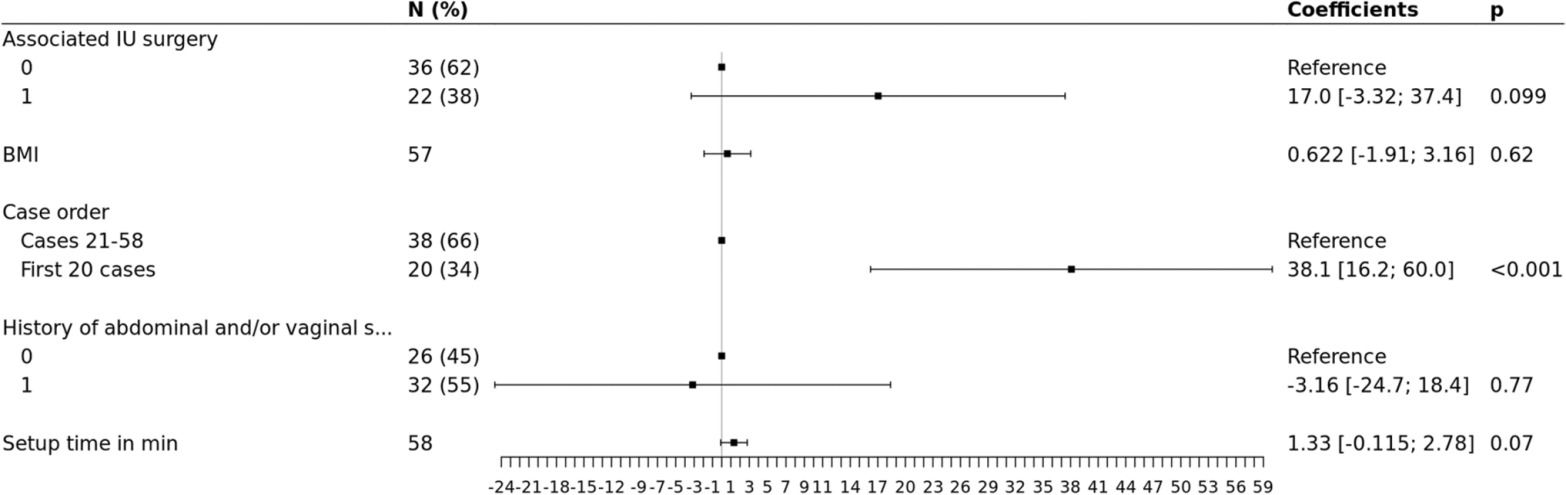
Forest plot of multivariate analysis of OOT difference within the learning curve, RASC group

Given that the RASC inclusions were consecutive cases early in the robotic promontofixation experience, we found interesting to study a potential difference of OOT in the learning curve. Indeed, adjusting for possible confounders, the mean OOT of the first 20 cases was superior of 38.1 min to the mean OOT of the later cases (*p* < **0.001**) (Figs. 4 and 5).

The complication rate was low for both groups, non-significantly different. Only 1 Clavien–Dindo stage 3 complication was found in this cohort, in the RASC group (1.7%), in a patient with concomitant Burch colposuspension, necessitating reoperation for hematoma evacuation in the Retzius space and at the same time, was also the only patient transfused. 3 complications involving the urinary system, 2 (3.4%) in RASC and 1 (2.4%) in the LASC group.

There was only one case needing laparotomy conversion, laparoscopic sacrocolpopexy being the original plan. Blood loss median estimation was 75 ml for RASC and 100 ml for LASC (*p* 0.18).

Mean hospital stay was 3.4 days for the RASC group (range 2–8) and 2.6 days (range 1–5) for the LSC group, a statistically significant difference (*p* < 0.01). This difference stays identical even if we don’t take into consideration the first 20 RASC interventions (3.4 vs. 2.6 days, *p* 0.004), considered as within the learning curve period and being conducted by the Si version using larger diameter ports, potential cause of more postoperative pain.

The anatomic result in the 6-week follow-up was described as poor in three RASC patients (8.6%) vs. one patient of the LSC group (4.5%), a non-statistically significant difference.

## Discussion

This is to our knowledge the first comparative study between RASC and LSC showing a statistically significant difference in total operative time in favor of the robotic group. (188 min vs 217, *p* < 0.01). The mean OOT of 188 min in our cohort is comparable with the mean 194 min of a meta-analysis in the subject cumulating 1488 patients [7]. What’s interesting, is that on the same meta-analysis, the mean OOT climbed to 265 min when only comparative studies RASC vs. LSC were included (6/27 studies, 219 vs. 224 patients, respectively), rendering the difference with LSC (mean OOT 206 min, *p* < 0.001) statistically significant. Special mention should be made for the study of Paraiso et al., the only RCT among them, including a 78 participants in total, with a stage II–IV posthysterectomy vaginal prolapse [8]. The authors published significantly longer median OOT in the RASC group (+ 67 min), compared with LSC and this, in every step of the procedure, including suture time (+ 31 min), for the same postoperative results. Although docking time could be considered responsible for longer OOT, the higher strict operative and suture times found in this study, come in contrast with our experience and tend to nullify the clear advantage of robotic assistance when deep dissection and multi-suturing are involved, raising the question of unequal familiarity in favor of the laparoscopic approach, on behalf of the surgeons, when the study was being conducted.

The only study included in this meta-analysis that showed a RASC advantage in terms of operative time, was a small-scale prospective study published by Seror et al. However, the difference was significant only for strict operative time, excluding robot setup and docking (median 125 min vs. 220; *p* < 0.0001), and erased when overall operating room time was taken into consideration (215 min vs. 220) [9]. We chose to compare the overall operating time for our primary results, as we consider it the most representative one, when we compare different surgical approaches. The specific for robotic surgery setup and docking times are also useful for secondary analysis. On the other hand, the strict operative time can have different definitions from study to study and does not reflect in any case the effective time of the surgery. Another time interval that we find in some publications is the overall operating room occupation time, which could, however, contain biases non-relevant to the surgical technique itself, like anesthesia or technical matters. In a normal situation, we do not find any reason why a patient undergoing robotic procedure should have longer presence in the operating room for any other reason than the overall operative time itself.

We used multivariate linear regression in an effort to limit possible confounders in the evaluation of our primary endpoint. For example, we know that obesity [10] or previous abdominal surgery are risk factors of difficult entry and higher operative difficulty in general, thus in any case, of prolonged OOT. While in the total study sample, OOT was significantly increased in obese and previous abdominal surgery patients (Fig. 2), these variables were not significantly related with OOT anymore, when investigated exclusively in the RASC group (Fig. 3). This could be another argument for seeking robotic assistance in the more difficult cases, to obtain maximal efficiency on its use.

In our secondary results, we found similar anatomic results in the 6 weeks postoperative visit with low failure rate for both groups. Although, the exact post-operative POP-Q values were not available for our study, a residual prolapse of POP-Q 2nd degree or more is in general conceived as a non-satisfactory postoperative result, in agreement with relevant literature [11]. Illiano et al. reach to the same conclusions in their recent RCT, with 100% correction of the apical compartment, with no difference in complication rate, perioperative bleeding and hospital stay, non-differently from the rest of the most important publications in the subject [12]. In our cohort, the hospital stay was significantly longer in the RASC group. Although not directly evaluated in our study, this could be explained by higher pain levels and more important narcotic use in early postoperative period in the RASC arm, possibly explained by higher abdominal pressures used in robotic procedures and feedback absence of robotic arms abdominal wall tension, as suggested by Anger et al. [13], leading us to the assumption that a more robot-specific ERAS protocol could be of value. That same RCT conducted by Anger et al. was sufficiently powered and had as primary objective to examine RASC cost-effectiveness. They found RASC not significantly more expensive than LSC, if buy and maintenance costs are deducted, contrary to previous publications, where the cost difference was mainly driven by the longer operation room occupation [8, 14]. By analogy, we could safely suggest that the shorter RASC operative time in our cohort, along with a regular use of the robot, should render the robotic alternative sufficiently cost-effective for this type of surgery.

Finally, we found a significant improvement in the mean OOT, after a learning curve of 20 RASC cases,, experience shared with other authors that have published a dramatic time improvement when they compared the first 20 procedures with the subsequent 127, in every step of the operation and of course in OOT [15]. Of note, in our series, this coincides with the Xi version upgrade, theoretically improving setup time due to its laser positioning device [16], although setup time was not a determinant factor (*p* 0.07) in the multivariate analysis (Fig. 5). Another previous publication, integrates to the learning curve the operation room staff and estimates the exact same number of cases needed, in order to be able to observe better setup times, even if in that study the subjects were undergoing robotic hysterectomy [17]. In our hospital, the choice was made to operate robotic surgery theater with a specially trained, non-rotating staff, and possibly, this is one of the reasons for the significant time save in our cohort.

## Conclusion

In conclusion, this study, despite the limitations of retrospective design and limited number of subjects, shows a clear interest of the robotic technology for this technically demanding intervention. We consider a strength of the study not only its monocentric design but also the fact that the same surgical team performed all the surgical procedures, limiting the risk of performance biases.

## Data Availability

The data that support the findings of this study are available from the corresponding author (EN), upon reasonable request.
